# Pulmonary epithelioid hemangioendothelioma presenting with vertebral metastases: a case report

**DOI:** 10.1186/1752-1947-8-201

**Published:** 2014-06-18

**Authors:** Angela Sardaro, Lilia Bardoscia, Maria Fonte Petruzzelli, Anna Nikolaou, Beatrice Detti, Giuseppe Angelelli

**Affiliations:** 1 Azienda Ospedaliero-Universitaria Policlinico di Bari, Dipartimento Interdisciplinare di Medicina, Sezione di Diagnostica per Immagini e Radioterapia, Università degli Studi di Bari “Aldo Moro”, Piazza Giulio Cesare 11, 70124 Bari, Italia; 2 Radioterapia, Azienda Ospedaliero-Universitaria Careggi, Largo Brambilla 3, 50134 Firenze, Italia

**Keywords:** Bone metastases, Endothelial markers, Epithelioid hemangioendothelioma, Radiation therapy, Vascular tumor

## Abstract

**Introduction:**

Epithelioid hemangioendothelioma is a rare vascular tumor that has an epithelioid and histiocytoid appearance, originates from vascular endothelial or pre-endothelial cells and comprises less than 1% of all vascular tumors. It was described for the first time in 1975 as pulmonary epithelioid hemangioendothelioma, because initially it was believed to be an aggressive form of bronchoalveolar cell carcinoma with a remarkable propensity to invade adjacent blood vessels and small airways. Only a few cases have been reported in the literature to date. Tumor cells expressing Fli-1 and CD31 have been identified as relatively specific endothelial markers. Epithelioid hemangioendothelioma may affect multiple organs and may vary considerably in its clinical and radiological presentation. More than 50% to 76% of pulmonary epithelioid hemangioendothelioma patients are asymptomatic. They are usually incidentally diagnosed on the basis of abnormal chest radiography during routine physical examinations. Hematologic and gastrointestinal disorders and weakness or numbness may also be observed, in addition to respiratory symptoms, in cases of disseminated pulmonary epithelioid hemangioendothelioma. Pain and swelling, pathological fractures, spine compression or paresthesia, loss of muscular strength and paraplegia may be present when bone metastases occur. Because of the rarity of this disease, there is no standard for treatment.

**Case presentation:**

A 46-year-old Caucasian woman presented to our institution in November 2009 with metastases of pulmonary epithelioid hemangioendothelioma from the L3 and L4 vertebrae. A course of radiotherapy at a dosage of 3,000cGy delivered in individual doses of 200cGy/day for 5 days/wk to the L3 and L4 vertebrae led to the disappearance of the patient’s lumbar pain without any detectable side effects. Percussion of the patient’s vertebral spine was negative, and no radiological progression of bone disease was found at her 1-year follow-up examination.

**Conclusion:**

Since epithelioid hemangioendothelioma was first correctly defined, several research groups have reported their experiences with epithelioid hemangioendothelioma irradiation. Further studies are needed to establish a standard radiation dose to be used for such a complex and extremely rare disease. In our present case, a radiotherapy dosage of 3,000cGy delivered in individual doses 200cGy/day for 5 days/wk allowed us to reach our goals: local pain control with good tolerance and better quality of life by the 1-year follow-up examination.

## Introduction

Representing less than 1% of all vascular tumors, epithelioid hemangioendothelioma (EHE) is a rare vascular tumor with an epithelioid and histiocytoid appearance that originates from vascular endothelial or pre-endothelial cells. It was described for the first time in 1975 by Dail *et al*. as *pulmonary epithelioid hemangioendothelioma* (PEH). Initially, it was believed to be an aggressive form of bronchoalveolar cell carcinoma with a remarkable propensity to invade adjacent blood vessels and small airways; hence the name *intra-vascular bronchioloalveolar tumor*[1].

The term *epithelioid hemangioendothelioma* was introduced in 1982 by Weiss and Enzinger in describing a vascular tumor of bone and soft tissue with intermediate malignancy and features between hemangioma and angiosarcoma [2]. Weldon-Linne *et al*., by using electron microscopy, confirmed previous findings about these tumor cells that they derive from a lineage capable of differentiation along endothelial lines. By immunohistochemistry, they revealed diffuse cytoplasmic staining of the malignant cells with a factor VIII–related antigen, which clarified the endothelial lineage of these tumor cells [3].

The World Health Organization 2002 classification [4] describes such tumors as lesions that fall into the category of locally aggressive tumors with metastatic potential. Therefore, some authors began to believe the hypothesis that EHE does not represent a distinct entity, but rather is an intermediate state of endothelial dedifferentiation with a highly variable and unpredictable prognosis [5].

Herein we present the case of a patient with extensive dissemination and serious bone damage due to PEH who benefited from radiation therapy (RT) for pain and control of local bone recurrence.

## Case presentation

A 46-year-old Caucasian woman presented to our institution in November 2009 with L3 and L4 vertebrae metastases of PEH. Previous thoracopulmonary investigations had revealed multiple disseminated pleuropulmonary nodules followed by pulmonary wedge resection, confirming the typical clinical PEH immunoreactivity for CD31, factor VIII, Fli-1 and vimentin. She had also undergone lumbar computed tomography (CT), which showed structural changes in the third and fourth lumbar vertebrae (L3-L4) combined with a partially collapsed L4 and L3-L4 disc protrusion. Her abdominal CT scan had shown a small, common iliac vein thrombosis and a small caval thrombosis immediately above the iliac venous confluence. All of these findings together led to initiation of therapy with polyethylene glycol and interferon α at a dosage of 1μg to 2μg once every 7 days. A CT scan performed 4 months after initiation of treatment revealed stable lung disease but new hypodense splenic lesions. A lumbar spine magnetic resonance imaging scan showed exacerbation of bone disease with worsening L3 and L4 collapse and further L4-L5 disc protrusion. Therefore, she underwent kyphoplasty. The bone biopsy performed during the operation confirmed the diagnosis of L4 vertebral body EHE.

When the patient presented to our institution, she complained of back pain that did not go away after taking common anti-inflammatory drugs and treatment with lumbar vertebrae acupressure. The pain was absent in other areas. On the basis of the scarce available literature on patients with this presentation, we delivered symptomatic RT into the L3-L5 vertebral tract using a 3,000cGy total target dose in 200cGy daily fractions 5 days/wk. At the end of the treatment, the patient’s lumbar pain had disappeared and she experienced no detectable side effects. In 2010, we observed negative percussion of the vertebral spine and no radiological progression of bone disease. The patient underwent ifosfamide and epirubicin chemotherapy and surgical removal of EHE spleen lesions soon after her follow-up examination at our institution. She died in October 2010 as a result of PEH progression.

## Discussion

After Dail *et al*. first identified PEH in 1975 [1], in 1982 Weiss and Enzinger used the term *epithelioid hemangioendothelioma* to describe a vascular bone and soft-tissue tumor with intermediate malignancy between hemangioma and angiosarcoma [2]. Weldon-Linne *et al*. discovered a factor VIII–related antigen in malignant cells, confirming their endothelial origin [3]. The median age of onset is 36 years (range, 20 to 60 years) [6]. The most common presentations are liver alone (21%), liver plus lung (18%), lung alone (12%) and bone alone (14%) [7]. Approximately 50% to 76% of patients are asymptomatic. Some present with chest pain, pleuritic pain, cough, dyspnea, malaise [8], hemoptysis and anemia. Bone metastases cause pain and swelling in the affected area, pathological fractures [9], spine compression if the lesions arise in vertebrae, which result in paresthesia, loss of muscular strength and paraplegia [10]. Hematologic and gastrointestinal disease, as well as weakness or numbness, may also occur because of EHE dissemination [7]. Radiologically, PEH consists of multiple bilateral perivascular nodular opacities with a diameter ≤1cm [10], and sometimes present with lymph node metastases, pleural thickening or ground-glass opacities. Bone metastases appear as osteolytic lesions with homogeneous contrast enhancement, cortical disruption and soft-tissue swelling [9]. Histologically, the tumor has a micropolypoid growth, and tumor cells with cytoplasmic vacuoles occasionally contain erythrocytes [11] expressing Fli-1 and CD31 [12], which are relatively specific endothelial markers. The overall survival rate of EHE patients is better when the disease is localized (90% at 1 year and 73% at 5 years) than when there is multi-organ progression (53% at 1 year and 24% and 5 years) [7].

Because of the rarity of this disease, there is no standard for treatment. Curative resection achieves good outcomes. Usually, adjuvant RT is used to control residual disease for patients with localized EHE. Chemotherapy (interferon 2α or carboplatin plus etoposide) is the preferred therapy for patients with widespread disease, but the benefits of these drugs are unclear [1,6,13].

Because of the radiobiological characteristics of PEH (slow growth), RT is ineffective. Good local control has been reported in a patient with exclusive EHE bone presentation treated by combining RT with bone surgery [14]. From the time EHE was correctly defined, several research groups have studied irradiation for EHE. A protocol of 4,000cGy for 4 weeks [13], followed by a 3,000cGy course of RT to the spine after surgical removal of EHE of the vertebrae [15] has been described. In the study reported by Aquilina *et al*., one patient survived for 11 years, but the other patients experienced worsening conditions due to multiple hepatic and abdominal metastases. Authors of another report described adjuvant RT delivered at dose of 6,400cGy to treat axillary EHE, which resulted in the absence of lymph node metastases but widespread pleuropulmonary metastasis [6]. Local irradiation after bone EHE resection at 6,000cGy in 23 fractions for 43 days showed good tolerance, without regional or distant metastases or local recurrence at 6-, 12- and 24-month follow-up examinations [10]. A recently reported protocol of 33 radiation fractions totaling 5,940cGy was applied to treat residual mastoid EHE. The authors found that imaging performed 8 years after surgery and RT revealed neither recurrence nor any change in the patient’s clinical status [16].

## Conclusion

Further studies are needed to establish the standard radiation dose to be used for locoregional control of this complex and extremely rare disease. In our present case, a RT dosage of 3,000cGy delivered at 200cGy/day 5 days/wk allowed us to reach our goals: local pain control with good tolerance and better quality of life at the 1-year follow-up examination.

## Consent

Written informed consent was obtained from the patient’s next of kin for publication of this case report and any accompanying images. A copy of the written consent is available for review by the Editor-in-Chief of this journal.

## Abbreviations

EHE: Epithelioid hemangioendothelioma; PEH: Pulmonary epithelioid hemangioendothelioma; RT: Radiation therapy (Radiotherapy).

## Competing interests

The authors declare that they have no competing interests.

## Authors’ contributions

AS analyzed and interpreted the patient data regarding the physical, radiological and histological examinations, elaborated the radiation treatment plan and was a major contributor to the writing of the manuscript. LB reviewed the available literature. MFP and AN assembled this report. BD and GA supervised the work. All authors read and approved the final manuscript.
